# O-GlcNacylation Links TxNIP to Inflammasome Activation in Pancreatic β Cells

**DOI:** 10.3389/fendo.2019.00291

**Published:** 2019-05-21

**Authors:** Gaelle Filhoulaud, Fadila Benhamed, Patrick Pagesy, Caroline Bonner, Yann Fardini, Anissa Ilias, Jamileh Movassat, Anne-Françoise Burnol, Sandra Guilmeau, Julie Kerr-Conte, François Pattou, Tarik Issad, Catherine Postic

**Affiliations:** ^1^INSERM U1016, Institut Cochin, Paris, France; ^2^CNRS UMR 8104, Paris, France; ^3^Université Paris Descartes, Sorbonne Paris Cité, Paris, France; ^4^Pasteur Institute de Lille, Lille, France; ^5^INSERM U1190 - EGID, Lille, France; ^6^UMR8251-CNRS, Paris, France; ^7^Université Paris Diderot, Sorbonne Paris Cité, Paris, France; ^8^CHU, Lille, France

**Keywords:** O-GlcNAcylation, TXNIP (thioredoxin-interacting protein), pancreatic beta cells, hyperglycemia, inflammasome

## Abstract

Thioredoxin interacting protein (TxNIP), which strongly responds to glucose, has emerged as a central mediator of glucotoxicity in pancreatic β cells. TxNIP is a scaffold protein interacting with target proteins to inhibit or stimulate their activity. Recent studies reported that high glucose stimulates the interaction of TxNIP with the inflammasome protein NLRP3 (NLR family, pyrin domain containing 3) to increase interleukin-1 β (IL1β) secretion by pancreatic β cells. To better understand the regulation of TxNIP by glucose in pancreatic β cells, we investigated the implication of O-linked β-N-acetylglucosamine (O-GlcNAcylation) in regulating TxNIP at the posttranslational level. O-GlcNAcylation of proteins is controlled by two enzymes: the O-GlcNAc transferase (OGT), which transfers a monosaccharide to serine/threonine residues on target proteins, and the O-GlcNAcase (OGA), which removes it. Our study shows that TxNIP is subjected to O-GlcNAcylation in response to high glucose concentrations in β cell lines. Modification of the O-GlcNAcylation pathway through manipulation of OGT or OGA expression or activity significantly modulates TxNIP O-GlcNAcylation in INS1 832/13 cells. Interestingly, expression and O-GlcNAcylation of TxNIP appeared to be increased in islets of diabetic rodents. At the mechanistic level, the induction of the O-GlcNAcylation pathway in human and rat islets promotes inflammasome activation as evidenced by enhanced cleaved IL1β. Overexpression of OGT in HEK293 or INS1 832/13 cells stimulates TxNIP and NLRP3 interaction, while reducing TxNIP O-GlcNAcylation through OGA overexpression destabilizes this interaction. Altogether, our study reveals that O-GlcNAcylation represents an important regulatory mechanism for TxNIP activity in β cells.

## Introduction

Chronic exposure to high glucose exerts deleterious effects on pancreatic β cell function leading to a disruption of their secretory capacities and/or a decrease in their cellular mass. The mechanisms driving β cells destruction are numerous including increased fatty acid cellular content, reactive oxygen species (ROS) production, macrophage infiltration, inflammation processes, and/or increased flux through the hexosamine biosynthetic pathway. Over the past years, Thioredoxin interacting protein (TxNIP) has emerged as a major mediator of β cell dysfunction, being one of the most up-regulated genes in response to hyperglycemia (1–3). As part of a negative-feedback loop, TxNIP inhibits glucose uptake and promotes caspase-1 cleavage, contributing to glucose-dependent β-cell death (2–6). TxNIP also regulates pro-inflammatory processes through the inflammasome activation *via* binding to NLRP3 (NOD-like receptor family pyrin domain containing 3) (7, 8). In this context, TxNIP is an important actor of pancreatic β cell biology and its tight regulation appears necessary for β cell survival.

The mechanisms driving TxNIP expression involve a crosstalk between several transcription factors. The *Txnip* promoter contains two carbohydrate response elements (ChoRE) for binding of the glucose sensitive transcription factor Carbohydrate Responsive Element Binding Protein (ChREBP) (2). While the Forkhead boxO1 transcription factor (FoxO1) was reported to up-regulate *Txnip* expression in neurons and endothelial cells, it was shown to significantly decrease its expression in pancreatic β cells. Mechanistically, FoxO1 was reported to prevent the glucose-induced *Txnip* expression by reducing the glucose-induced binding of ChREBP at the promoter, suggesting that FoxO1 competes with ChREBP for binding to the *Txnip* promoter. The TxNIP protein is also regulated at the posttranslational level through phosphorylation (9). In the current study, we addressed whether the TxNIP protein could be regulated through O-GlcNAcylation, a posttranslational modification that depends on intracellular glucose flux through the hexosamine biosynthetic pathway. O-GlcNAcylation, which is linked to glucotoxicity in many cell types, modulates protein activity and/or partner interactions (10, 11). O-GlcNAcylation requires the activity of two enzymes: the O-GlcNAc transferase (OGT), which transfers the monosaccharide to serine/threonine residues on target proteins, and the O-GlcNAcase (OGA), which hydrolyses this sugar.

Our study demonstrates that the TxNIP protein is modified by O-GlcNAcylation in both rodent and human pancreatic β cells and that this modification enhances its interaction with its binding partner NLRP3, leading in turn to inflammasome activation.

## Research Design and Methods

### Animals

Animal experiments were performed in agreement with protocols approved by French guidelines. Eight week-old male C57BL/6J and *db/db* mice were purchased from Harlan®. Mice were adapted to the environment for 1-week prior to study and maintained in a 12-h light/dark cycle with water and regular diet (65% carbohydrate, 11% fat, and 24% protein). When specified mice were fasted for 24 h and then refed for 18 h with a high carbohydrate diet (72.2% carbohydrate, 1% fat, 26.8% protein). Ten weeks-old male Wistar rats were purchased from Harlan®. Goto-Kakizaki (GK) rats were obtained from the GK/Par colony obtained from the Movassat's laboratory (12).

### Isolation of Islets of Langerhans From Rodent Models

Islets of Langerhans were obtained from 3 months old Wistar and Goto-Kakizaki (GK) rats by collagenase digestion and Ficoll gradient and then hand-picked as described previously (13). Freshly isolated islets were cultured in 6 wells plates and incubated in 5.5 or 16.7 mM glucose in the absence or presence of 100 μM PUGNAc (Sigma) for 72 h in RPMI 1640 supplemented with 10% fetal calf serum, 100 U/ml penicillin, 100 mg/ml streptomycin and 10 mM L-glutamine.

### Culture and Transfection Experiments in HEK293

Human embryonic kidney cells (HEK293) were grown in 6 wells plates in 25 mM D-glucose DMEM supplemented with 10% fetal calf serum (Sigma®). The OGT and OGA plasmids were previously described (10), the TxNIP plasmid was purchased from Genecust® and pcDNA3-N-Flag-NLRP3 was a gift from Bruce Beutler (Addgene plasmid # 75127). Transfections of HEK293 cell were performed using Lipofectamine 2000 and OptiMEM, and 1 μg of plasmid/well.

### Culture and Transfection Experiments in INS1 832/13 Cells

INS1 832/13 cells (kindly provided by Dr. CB Newgard, Duke University Medical Center, Durham, NC) were cultured in RMPI 1640 supplemented with 10% fetal calf serum (Life Technology®), 100 U/ml penicillin, 100 mg/ml streptomycin, 1 mM sodium pyruvate, 10 mM HEPES and β-mercaptoethanol. Cells were then washed, starved during 6 h in 2.5 mM glucose without serum and further incubated with 2.5 or 20 mM glucose during 24 h. INS1 832/13 cells were infected with shOGT, shcontrol, GFP (Genecust®) and OGA adenoviruses (a kind gift from Dr. Xao Yang) during 24 h. Cells were then washed, starved during 6 h in 2.5 mM glucose without serum and stimulated in 5 or 25 mM during 24 h.

For TxNIP reporter assays, INS1 832/13 cells were transfected with a Txnip luciferase reporter (a promoter containing the two tandem ChoRE) and a plasmid expressing β Galactosidase (0.2 μg DNA of each plasmid per well) using Lipofectamine 2000. β Galactosidase assays were performed for normalization of the ChoRE luciferase activity. The luciferase assay was conducted using the dual luciferase substrate system (E1501; Promega, Madison, WI), and the result was normalized with the internal control *Renilla luciferase*. Each experiment was repeated at least three times.

For Bioluminescence Resonance Energy Transfer (BRET) experiments, INS1 832/13 cells were transfected with the cDNA coding for a biosensor (14, 15) based on BRET, in which the precursor pro–IL1β is fused at its terminals to RLuc8 (a variant of *Renilla* luciferase) and Venus (a variant of yellow fluorescent protein). Forty-eight hours after transfection BRET measurements were performed as described previously (16). Results are expressed in milliBRET units as defined previously (17).

### Isolation, Culture, and Analysis of Human Islets

Human islets were isolated from pancreata harvested from adult brain-dead individuals in the context of the traceability requirements for our clinical islet transplantation program (clinicaltrials.gov, NCT01123187, NCT00446264, NCT01148680) as described previously (18). The experimental design was approved in agreement with French regulations, our Institutional Ethical Committee of the University of Lille and the Center Hospitalier Régional Universitaire de Lille. Islets were allowed to recover in culture after isolation for at least 18 h before cell treatments. For experiments investigating glucose dependence of TxNIP, OGT, and OGA mRNA and protein levels, human islets were cultured in glucose-free medium (Gibco, Life Technologies, Paris, France) supplemented with 10% FBS, 1% P/S, 15 mM HEPES, and 5.5 or 16.7 mM glucose with and without PUGNAc (100 μM). Pellets were harvested for RNA and protein analysis. Total RNA was extracted using the RNeasy Mini Kit (Qiagen, Courtabœuf, France) (19).

### Quantitative Real-Time PCR

Total RNA was extracted using RNeasy micro Kit (Qiagen®) for rodent islets and RNeasy Kit (Promega®) for INS1 832/13 cells. cDNA was reversed transcribed. The levels of expression of each gene were normalized to cyclophylin expression (INS1832/13 and rat islets) and β-actin and cyclophylin (human islets).

### Western Blotting Analysis

Proteins from rodent and human islets and cell lines were subjected to 10% SDS-PAGE and transferred to nitrocellulose membranes. Rabbit polyclonal NLRP3 (Cell signaling), OGT (Sigma), cleaved IL1β (Cell Signaling) and monoclonal TxNIP (MBL) antibodies were used. O-GlcNAc was detected using RL2 anti-OGlcNAc antibody (Abcam). HSP90 (Cell Signaling), GAPDH (Cell Signaling), and β-actin were used to normalize data as indicated on Figure legends.

### Immunoprecipitation and Wheat Germ Agglutinin Purification

For TxNIP immunoprecipitation, cells were lysed on IPH buffer (20 mmol/L Tris/HCl, 150 mmol/L NaCl, 0.5% NP-40 [v/v], and protease inhibitors). Proteins were incubated with 2 μg of anti-TxNIP antibody (MBL) and placed at 4°C overnight. Bound proteins were recovered after addition of 30 μl of Sepharose-labeled protein G (Sigma) for 1 h at 4°C. Beads were gently centrifuged for 1 min and washed four times for 5 min each. Bound proteins were analyzed by Western blot with a polyclonal anti-Flag (Sigma) or NRLP3 (Cell signaling) antibodies. For wheat germ agglutinin ([WGA] a GlcNAc-binding lectin) precipitation, 1 mg of proteins was incubated with 30 μl of WGA agarose beads (Sigma). Then, proteins were eluted from the beads in a Laemmli buffer and separated by SDS-PAGE.

## Results

### TxNIP Expression and O-GlcNAcylation Are Increased in Pancreas and Islets of Rodent Models of Type 2 Diabetes

**Figure 1:**
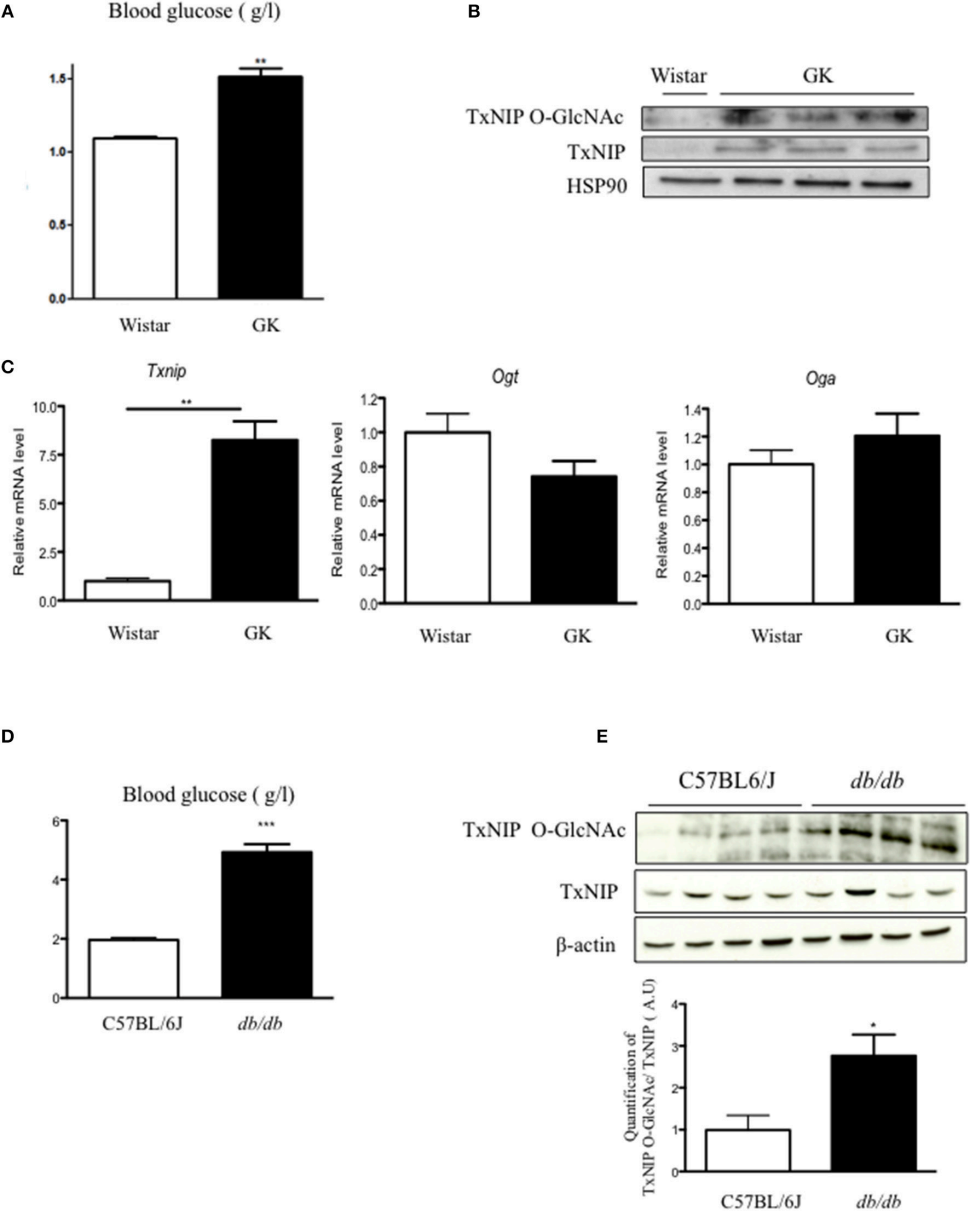
O-GlcNAcylation of TxNIP is increased in rodent models of diabetes. **(A)** Blood glucose concentrations were measured in fed condition in wister and GK rats. **(B)** *Txnip, Ogt*, and *Oga* mRNA levels in islets of wistar and GK rats. Significance is based on Mann-Whitney test. ^**^p < 0.01. *n* = 3–5. **(C)** TxNIP O-GlcNAcylation level evaluated by WGA binding experiments. Islet lysate proteins were immunoblotted with TxNIP and HSP90 was used as a loading control. **(D)** Blood glucose concentrations were measured in C57/B6J and *db/db* mice. Significance is based on Mann-Whitney test. ^***^p < 0.001 **(E)** TxNIP O-GlcNAcylation was evaluated by WGA binding experiments. Whole pancreas proteins were immunoblotted with a TxNIP antibody and β-actin was used as a loading control. Quantification of the ratio of O-GlcNAcylated TxNIP corrected to total TxNIP protein is shown. Significance is based on student's *T*-test followed by Welch correction *post-hoc* test. ^*^p < 0.05. *n* = 4.

We first evaluated TxNIP expression and O-GlcNAcylation in islets of GK rats, a diabetic but non-obese rat model with moderate hyperglycemia but severe β cell defect (20) (Figure 1). We confirmed that GK rats were hyperglycemic (Figure 1A) and showed that *txnip* mRNA expression was markedly increased (8-fold) (Figure 1B), while no significant modification in either *Ogt* or *Oga* expression was observed (Figure 1C). In control islets, TxNIP protein was barely detectable, neither in cell lysates nor on WGA eluates (Figure 1B). In contrast, TxNIP protein could be detected in cell lysates from GK islets. Recovery of TxNIP on WGA beads (Figure 1C) suggested that TxNIP was O-GlcNAcylated in rat GK islets.

We next examined TxNIP protein content and O-GlcNAcylation of TxNIP (TxNIP O-GlcNAc) in pancreas from *db/db* mice. Blood glucose measurement confirmed the marked hyperglycemia of *db/db* mice vs. C57BL/6J mice (Figure 1D). Although total TxNIP protein content was unchanged in pancreas from *db/db* mice, the amount of TxNIP immunoprecipitated on WGA beads was higher in *db/db* mice than in control C57BL/6J mice. Densitometric analysis of the signals revealed a 3-fold increase in the TxNIP O-GlcNAc/TxNIP ratio (Figure 1E). Taken together, our data suggest that TxNIP is O-GlcNAcylated in pancreas of diabetic rodents.

### O-GlcNAcylation of TxNIP Depends on OGT Activity

**Figure 2:**
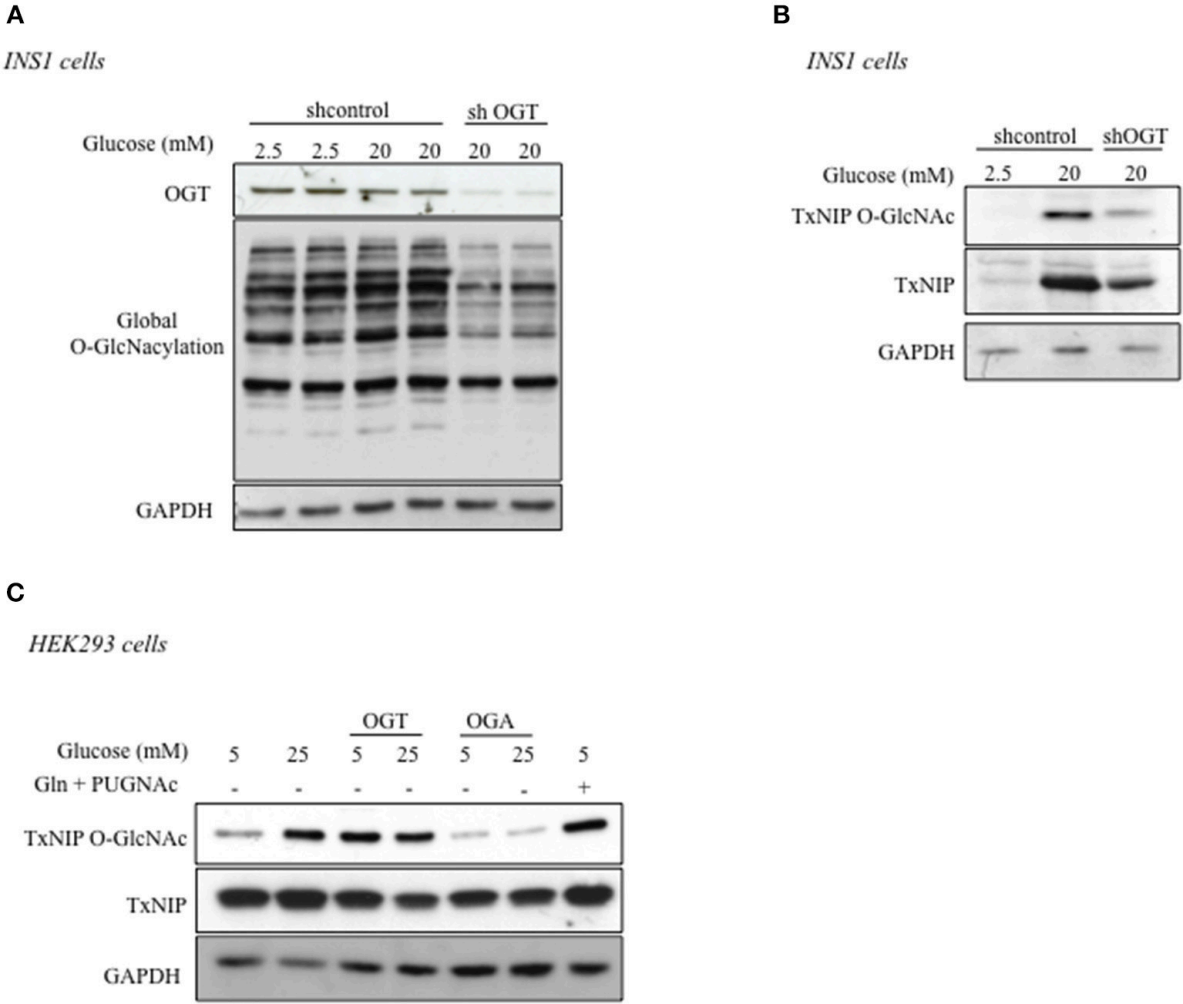
TxNIP O-GlcNAcylation is dependent on OGT. **(A,B)** INS1 832/13 cells were infected by a shControl or shOGT adenovirus for 24 h. INS1 832/13 cells were later stimulated for 24 h under low glucose (2.5 mM) or high glucose (20 mM) concentrations. **(A)** Global O-GlcNAcylation levels and OGT protein content. GAPDH was used as a loading control. **(B)** TxNIP O-GlcNAcylation was evaluated by WGA binding experiments. Protein lysates from INS1 832/13 cells were immunoblotted with TxNIP and GAPDH was used as a loading control. **(C)** HEK293 cells were co-transfected with TxNIP, OGT, and OGA plasmids and incubated for 24 h under low (5 mM) or high glucose conditions (25 mM) supplemented or not by PUGNAc and glucosamine (Gln). O-GlcNAcylation level of TxNIP was evaluated using an anti-O-GlcNAc antibody (RL2). Immunoprecipitation (IP) of TxNIP was analyzed by immunoblotting with a Flag antibody. Representative Western blot from *n* = 3 experiments for TxNIP protein content are shown. GAPDH was used as loading control.

To characterize the role of OGT in the regulation of TxNIP O-GlcNAcylation, the enzyme was silenced through a shRNA approach in INS1 832/13 cells (Figures 2A,B). We first verified the efficiency of the shRNA to knock down OGT expression and global protein O-GlcNAcylation in these cells. As shown in Figure 2A, OGT silencing under high glucose concentrations (20 mM) led to a decrease in O-GlcNAcylated proteins levels compared to shcontrol conditions. We then examined, under shOGT conditions, TxNIP O-GlcNAcylation by WGA precipitation. We observed that TxNIP recovery on WGA beads was markedly reduced compared to shcontrol conditions. However, total TxNIP protein content was also reduced when OGT was silenced (Figure 2B). Therefore, to evaluate TxNIP O-GlcNAcylation independently of any change in TxNIP expression, we transfected HEK293 cells with a TxNIP expression plasmid together with an OGT or OGA plasmid (Figure 2C). TxNIP was then immunoprecipitated and its O-GlcNAcylation level was evaluated using an anti-O-GlcNAc antibody in HEK293 cells cultured under low (5 mM) or high (25 mM) glucose concentrations, or under low glucose supplemented with glucosamine (Gln, 5 mM) and PUGNAc (an inhibitor of OGA activity). We observed that TxNIP O-GlcNAcylation increased in response to 25 mM glucose and was reduced when OGA was overexpressed. Moreover, TxNIP O-GlcNAc markedly increased when OGT was overexpressed under 5 mM glucose. Altogether, these results show that TxNIP is O-GlcNAcylated in response to high glucose concentrations and that this modification depends on OGT activity.

### Increased TxNIP O-GlcNAcylation Promotes Inflammasome Activation in Human and Rat Islets

**Figure 3:**
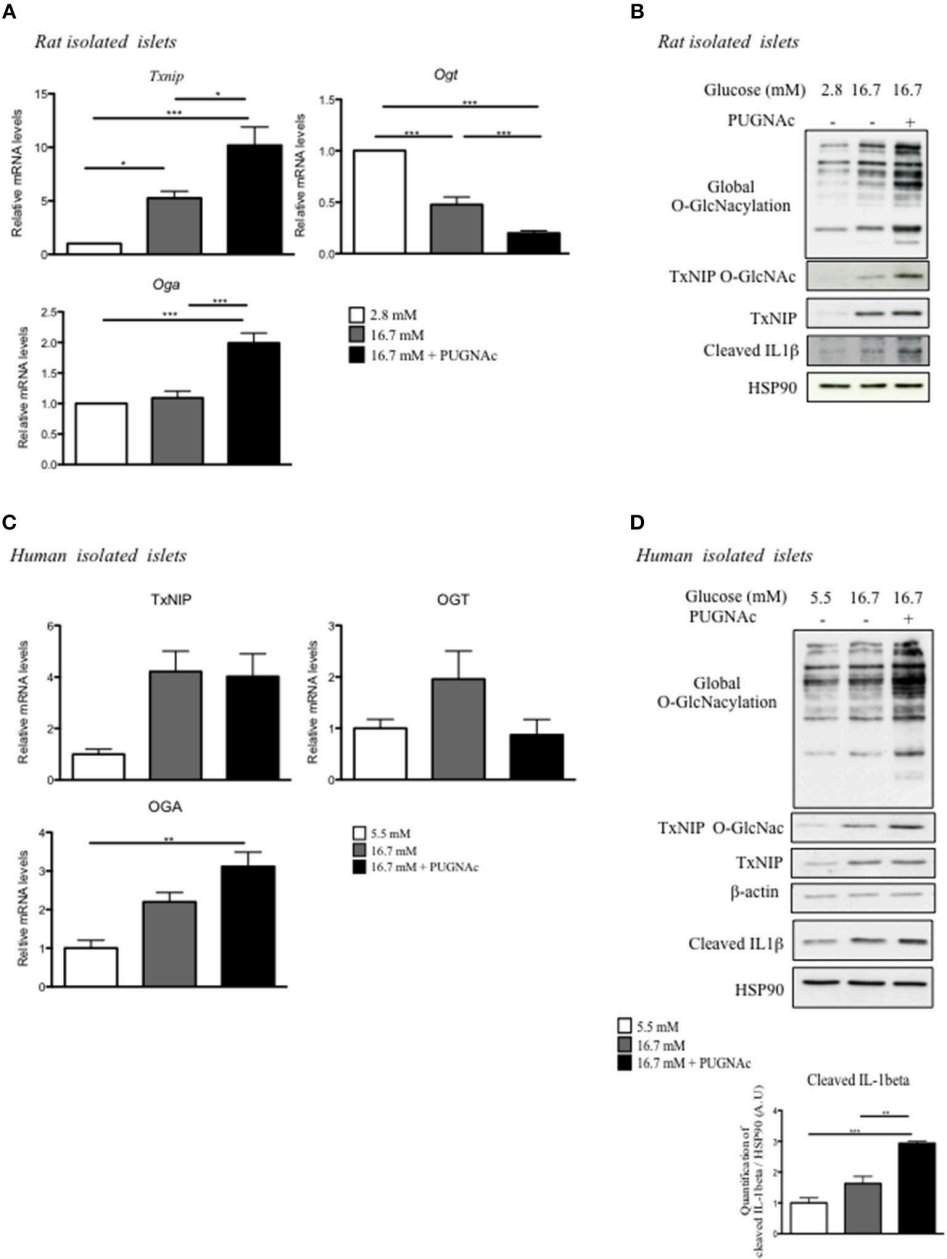
Increasing O-GlcNAcylation in rat and human islets promotes inflammasome activation. **(A,B)** Islets isolated from Wistar rat were incubated for 72 h under low glucose (2.8 mM), high glucose (16.7 mM), or high glucose plus PUGNAc (16.7 mM + PUGNAc) **(A)** Q-PCR analysis of OGT, OGA, and TxNIP. Data are means ± SEM of eight independent experiments. Significance is based on two-way ANOVA followed by a Bonferroni *post-hoc* test ^*^p < 0.05, ^**^p < 0.01, ^***^p < 0.001. **(B)** Global O-GlcNAcylation levels and TxNIP protein content. TxNIP O-GlcNAcylation was evaluated by WGA binding experiments. Representative Western blot for cleaved Il1β protein content is shown. HSP90 was used as a loading control. **(C,D)** Human islets were incubated for 72 h under low glucose (5.5 mM), high glucose (16.7 mM), or high glucose plus PUGNAc (16.7 mM + PUGNAc) **(C)** QPCR analysis of TxNIP, OGT, and OGA mRNA expression. Data are means ± SEM. *n* = 3 independent experiments. Significance is based on two-way ANOVA followed by a Bonferroni *post-hoc* test ^**^p < 0.01. **(D)** Global O-GlcNAcylation levels, TxNIP protein content, and O-GlcNAcylation of TxNIP evaluated by WGA binding experiments. β-actin was used as a loading control. Representative Western Blot of Cleaved Il1β and HSP90 are also shown. Data are means ± SEM. *n* = 3 independent cultures. Significance is based on two-way ANOVA followed by a Bonferroni *post-hoc* test ^**^p < 0.01, ^***^p < 0.001.

To better understand the regulation of TxNIP by O-GlcNAcylation, we performed a series of experiments *ex vivo* in response to glucose with or without PUGNAc (Figure 3). The regulation of TxNIP by O-GlcNAcylation was examined in isolated rat islets cultured in 2.8 mM glucose, 16.7 mM glucose, or 16.7 mM glucose supplemented with PUGNAc (Figures 3A,B). As previously reported in other cell types (21, 22), treatment of rat islets with the OGA inhibitor PUGNAc increased *Oga* and decreased *Ogt* mRNA levels (Figure 3A). *Txnip* mRNA expression, protein levels and O-GlcNAcylation were significantly induced in response to elevated glucose concentrations (a 5-fold increase when comparing 2.8–16.7 mM glucose concentrations) and was further increased (a 2-fold increase) when PUGNAc was present (Figure 3A). Using a luciferase reporter gene under the control of *Txnip* promoter (containing the two tandem ChoRE), we confirmed transcriptional regulation by glucose in pancreatic beta cells (Supplementary Figure 1). Indeed, in INS1 832/13 cells, high glucose concentration (20 mM) stimulated by about 30-fold *Txnip* promoter activity compared to low glucose concentrations (5 mM). Interestingly, *Txnip* promoter activity was reduced by 50% when INS1 832/13 cells were cultivated under high glucose concentrations and infected by the OGA adenovirus (Supplementary Figure 1).

A marked increase in TxNIP total protein content was also observed in rat islets cultured in presence of 16.7 mM when compared to 2.8 mM glucose, and a further increase in total TxNIP protein content could be detected in presence of PUGNAc. O-GlcNAcylated forms of TxNIP were enhanced and paralleled with global O-GlcNAcylation of proteins under glucose conditions with or without PUGNAc (Figure 3B).

We also cultured human islets for 48 h in glucose 5.5, 16.7, or 16.7 mM supplemented with PUGNAc and analyzed for expression and O-GlcNAcylation of TxNIP (Figures 3C,D). We observed a 4-fold induction of *TxNIP* expression in response to elevated glucose concentrations (16.7 mM). Adding PUGNAc to high glucose concentration did not further increase *TxNIP* expression. *OGA* expression was upregulated in human islets cultured under high glucose concentrations and PUGNAc whereas no difference in *OGT* expression was observed (Figure 3C). In agreement with *TxNIP* mRNA expression, TxNIP protein content was increased with 16.7 mM glucose and adding PUGNAc did not modify total TxNIP protein content but increased its O-GlcNAcylated form as well as global O-GlcNAcylation levels (Figure 3D). Since a link between TxNIP and the inflammasome was previously evidenced (23), we measured cleaved IL1β under glucose ± PUGNAc conditions. Human islets cultured with 16.7 mM glucose exhibited a 1.6-fold increase in cleaved IL1β and supplementation of the culture medium with PUGNAc led to a further 2.9-fold increase, suggesting potentiation of inflammasome activation (Figure 3D). Similarly to human islets, a significant increase in cleaved IL1β was observed in response to 16.7 mM glucose and PUGNAC conditions in rat islets (Figure 3B).

### TxNIP O-GlcNAcylation Modifies Its Scaffold Function With NLRP3

**Figure 4:**
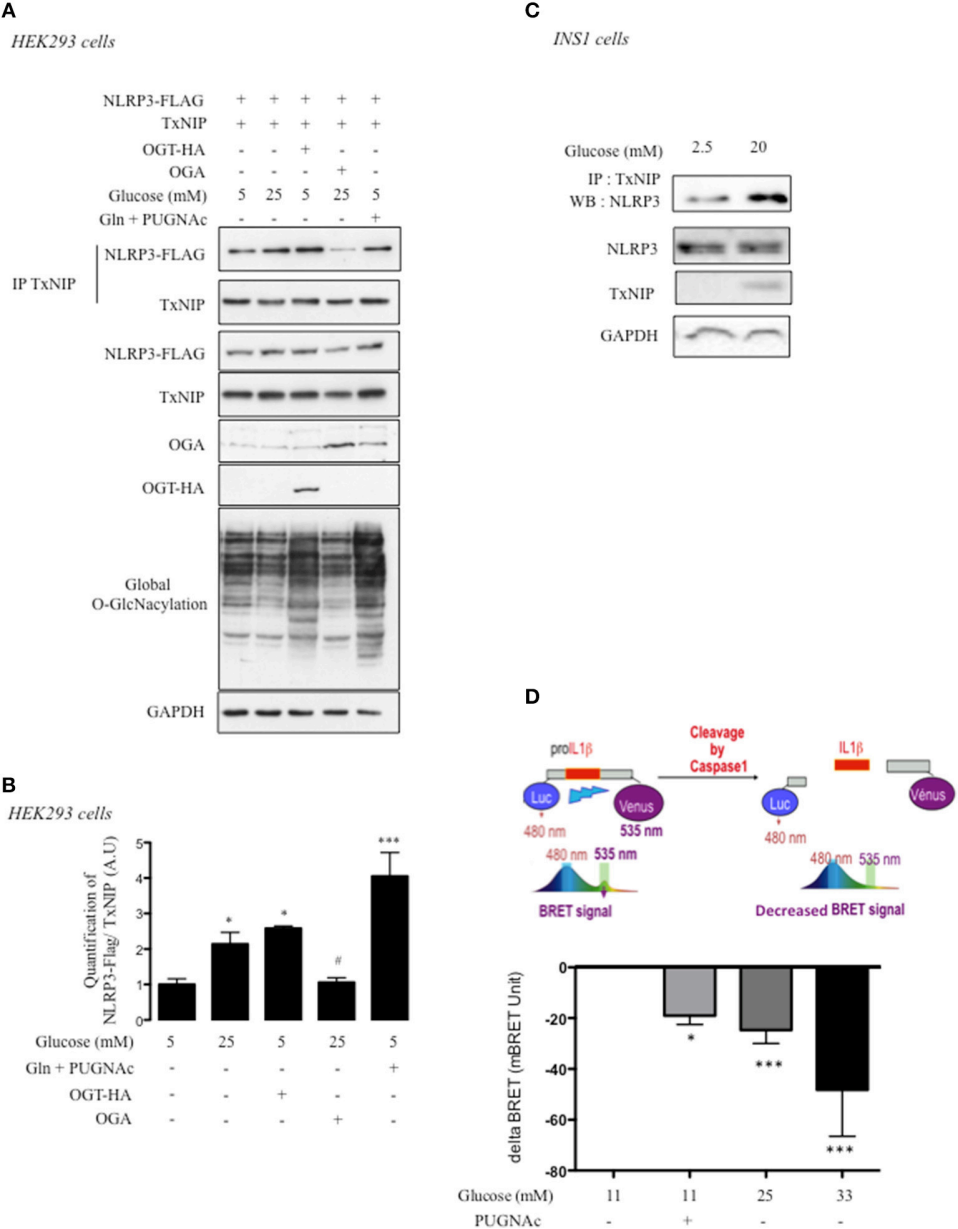
O-GlcNAcylation promotes TxNIP interaction with NLRP3 and pro-Il1β cleavage. **(A)** HEK293 cells were co-transfected with TxNIP, NLRP3-flag, OGT, and OGA plasmids and incubated for 24 h under low glucose condition (5 mM), high glucose condition (25 mM), or low glucose condition supplemented with PUGNAc and glucosamine (Gln). Immunoprecipitation (IP) of TxNIP was analyzed by immunoblotting with a Flag antibody. Representative Western blot for NLRP3, TxNIP, OGA, and OGT protein content are shown *n* = 4–7 independent experiments. GAPDH was used as loading control. **(B)** Quantification of the interaction between TxNIP and NLRP3 normalized to total TxNIP protein. Significance is based on two-way ANOVA followed by a Bonferroni *post-hoc* test. ^*^p < 0.05, ^***^p < 0.001 when compared to 5 mM glucose; ^#^*p* < 0.05 when compared to 25 mM glucose. **(C)** INS1 832/13 cells were stimulated for 24 h under low glucose (2.5 mM) or high glucose (20 mM) concentrations supplemented with PUGNAc. Immunoprecipitation (IP) of TxNIP was analyzed by immunoblotting with a NLRP3 antibody. Representative Western blot for NLRP3 and TxNIP protein content are shown. GAPDH was used as loading control (*n* = 4–7 independent experiments). **(D)** INS1 832/13 cells were transfected with a BRET-based biosensor that monitors pro-Il1β cleavage. The histogram shows the decreased in BRET signal measured in INS1 832/13 cells after 24 h of incubation with PUGNAc or with 25 mM or 33 mM of glucose. Significance is based on two-way ANOVA followed by a Dunnett's test for BRET experiments. ^*^p < 0.05, ^***^p < 0.001 (*n* = 4–14).

TxNIP functions as a scaffold protein and interacts with different partners to inhibit or activate their biological activities (24). Therefore, we next addressed whether O-GlcNAcylation of TxNIP could affect IL1β cleavage through its interaction with NLRP3. Experiments were performed in both HEK293 and INS1 832/13 cells (Figure 4). HEK293 cells were co-transfected with a TxNIP expression vector and a NLRP3 expression vector tagged with a FLAG epitope (NLRP3-FLAG). Immunoprecipitation of cell lysates with a TxNIP antibody and immunoblotting with anti-Flag antibody revealed that TxNIP co-immunoprecipitated with NLRP3 (Figure 4A). A 2-fold increase in the interaction between TxNIP and NLRP3 was observed under high glucose (25 mM) compared to low glucose concentrations (5 mM) (Figures 4A,B). OGT overexpression under low glucose concentrations induced a level of interaction similar to that obtained under high glucose conditions alone (Figures 4A,B). Inhibition of O-GlcNAcylation *via* OGA overexpression significantly decreased the interaction between the two partners. Finally, stimulation of O-GlcNAcylation with glucosamine (Gln) and PUGNAc led to a greater interaction between TxNIP and NLRP3 (Figures 4A,B).

Interaction between endogenous TxNIP and NLRP3 proteins was also evidenced in INS1 832/13 cells upon immunoprecipitation with anti-TxNIP antibody and immunobloting with anti-NLRP3 antibody (Figure 4C). Under high glucose concentrations (20 mM), the interaction between TxNIP and NLRP3 was increased compared to low glucose concentration (2.5 mM). Finally, we used a BRET approach in INS1 832/13 cells to address the importance of the O-GlcNAcylation modification for the activation of the inflammasome pathway. INS1 832/13 cells were transfected with a plasmid coding a BRET biosensor comprising the pro-Il1β sequence flanked by a Luciferase and an YFP (14). Cleavage of the pro-Il1β results in a decreased in BRET signal (Figure 4D). A significant decrease in BRET signal was observed when INS1 832/13 cells were cultured under 11 mM glucose with PUGNAc (Figure 4D), similar to that obtained with 25 mM glucose. Higher glucose concentrations (33 mM glucose) further decreased the BRET signal. Of note, the effect of PUGNAc on BRET signal was confirmed using Thiamet G, a more specific inhibitor of O-GlcNAcase activity (Supplemental Figure 2). Altogether, our results reveal that increased TxNIP O-GlcNAcylation correlates with the induction of the inflammasome pathway.

## Discussion

The current study demonstrates that TxNIP protein is modified by O-GlcNAcylation in pancreatic β-cells in an OGT dependent manner. We report here that this posttranslational modification, dependent on high glucose concentrations, increases the interaction of TxNIP with its partner NLRP3, correlating with enhanced cleavage of the interleukin IL1β in both rodent and human cells.

Over the past years, TxNIP has emerged as a central regulator of β-cell function. TxNIP is one of the most up-regulated gene in response to glucose in human islets and INS1 cells (2, 9, 25). We confirmed that *Txnip* promoter activity is markedly increased in response to glucose in INS1 cells, an effect previously reported to be ChREBP dependent (25). Overexpression of TxNIP in INS1 cells is associated with increased apoptosis (3, 26) while β-cell specific TxNIP inhibition protects against β cell dysfunction under high glucose concentrations by increasing β cell mass and stimulating the cellular survival pathway Akt/Bcl-xl. TxNIP is also implicated in the production of insulin by regulating miR-204 expression which in turn targets the transcription factor MafA that binds to the promoter in the *insulin* gene (27). While the regulation of TxNIP was essentially described at the transcriptional level, in particular by the transcription factors ChREBP and FoxO1 in pancreatic β cells (9), post-translational regulation of the TxNIP protein by phosphorylation was described in adipocytes and myotubes in response to insulin (28). Interestingly, in these cells, phosphorylation of TxNIP by AMPK or AKT leads to its dissociation from glucose transporters and to its degradation thereby enhancing glucose uptake (29, 30).

While experiments from Ayer's laboratory previously showed the importance of the hexosamine biosynthetic pathway for the regulation of TxNIP (31), a regulation of TxNIP protein by O-GlcNAcylation had never been described to our knowledge. In the present work, we provide several lines of evidence in favor of TxNIP O-GlcNAcyaltion. It is important to note that although binding of a protein to WGA does not necessarily prove that this protein is O-GlcNAcylated, the experiments we performed in HEK cells in which a transfected Flag-TxNIP was immunprecipitated and probed with an anti-O-GlcNAc antibody, strongly supports the fact that the TxNIP protein is indeed modified by O-GlcNAcylation. Similar experiments with the endogenous TxNIP in pancreatic β-cells, were problematic given that TxNIP expression was also increased upon induction of O-GlcNAcylation in beta cell, thereby limiting clear interpretation. O-GlcNAcylation, which involves the addition of a single O-GlcNAc to serine and threonine residues of proteins acts as a nutrient sensor. A couple of enzymes is involved in the regulation of the pathway: OGT, the enzyme which transfers the mono- saccharide to serine/threonine residues on target proteins, and OGA, which hydrolyses the sugar. Several studies have established that inhibition of OGA activity and subsequent increase in O-GlcNAcylation result in an enhanced OGA mRNA and protein expression (22, 32–34), probably as an adaptive mechanism to maintain O-GlcNAc homeostasis in the cell. In agreement with this notion, several lines of evidence indicated that in patients with diabetes, increased O-GlcNAcylation associated with chronic hyperglycaemia was also associated with an increased expression of OGA (35, 36). Interestingly, in a recent study (37) OGA mRNA levels in leucocytes from patients with type 2 diabetes were significantly correlated with TxNIP mRNA levels, as well as with blood markers of hyperglycaemia (HbA1C, Fructosamine). We report here that OGA mRNA levels are increased in response to enhanced O-GlcNAcylation pathway in both human and rat islets. The regulation of OGT by O-GlcNAc homeostasis is less clear. In rat islets, we observed that OGT expression is negatively regulated in response to high glucose concentrations and that this inhibition is stronger when the OGA enzyme is blocked by PUGNAc. Interestingly, OGT expression was not modified by the different treatments in human islets suggesting a species dependent regulation.

Several transcription factors are regulated by O-GlcNAcylation in pancreatic β cells (11). For example, O-GlcNAcylation of FoxO1 in INS1 cells results in a 2-fold increase in its transcriptional activity and a 3-fold increase in the expression of the insulin-like growth factor-binding protein 1 (*Igfbp1*) gene at the mRNA level, resulting in IGFBP1 protein hypersecretion by INS1 cells. In turn, increased IGFBP1 production in the culture medium blunts the Akt transduction pathway, revealing a novel mechanism by which O-GlcNAcylation inhibits Akt activity in INS1 cells through an autocrine mechanism (38). Of note, O-GlcNAcylation not only regulates transcriptional activity but has also been shown to influence protein-protein interactions (39, 40). In the current study, we revealed a novel mechanism by which O-GlcNAcylation of TxNIP favors its interaction with its partner NLRP3. NLRP3 is an inflammasome protein that once activated leads to the processing of IL1β, a cytokine involved in the pathology of type 2 diabetes (41). By performing both Western blot and BRET analysis, we demonstrated that production of IL1β by pancreatic cells could be induced by both glucose and PUGNAc. Therefore, increased TxNIP O-GlcNAcylation in pancreatic islets could play a part in diabetes pathogenesis. In the current study, two rodent models of diabetes (GK rats and *db/db* mice) suggested O-GlcNAcylation of TxNIP in pancreatic cells. Of note, TxNIP expression level was increased in isolated islets from GK rats while no difference was observed in the *db/db* mouse model. The difference could be explained by the fact that we had access to isolated islets for GK rats but not for *db/db* mice.

**Figure 5:**
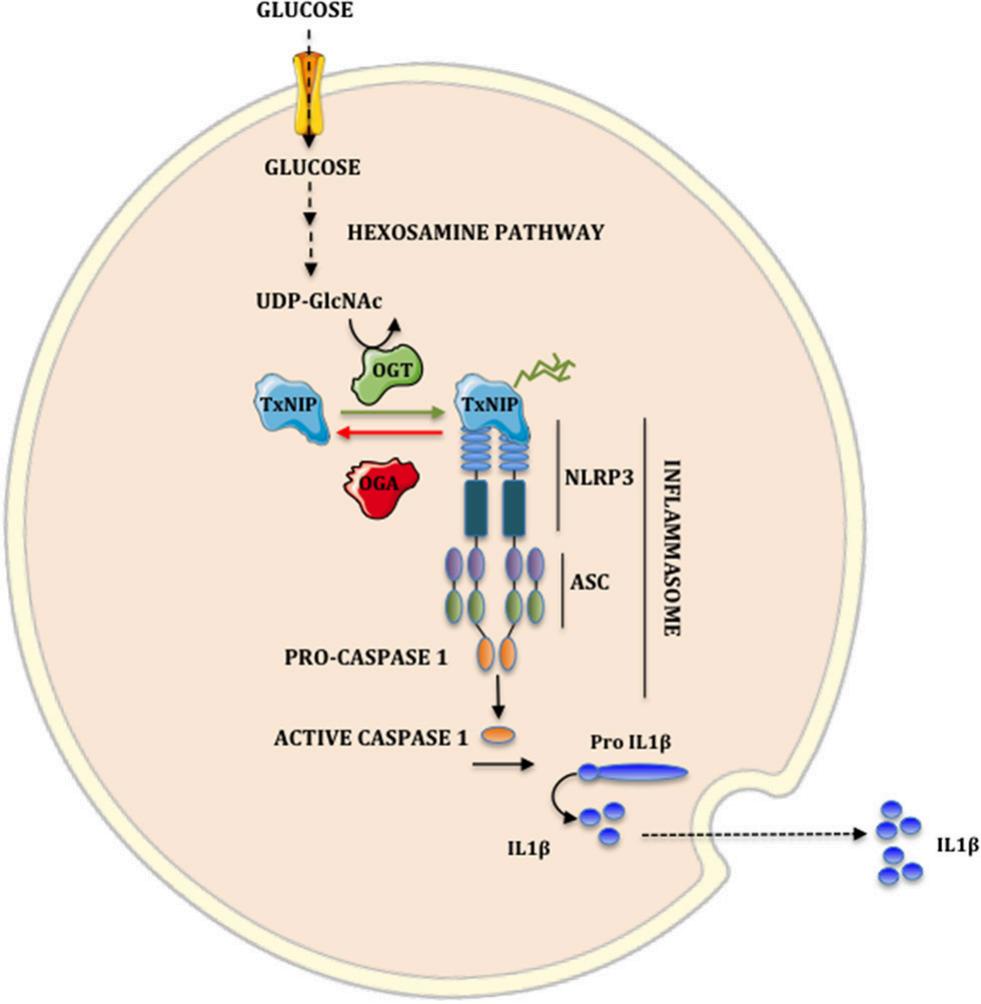
Implication of hexosamine biosynthetic pathway on the NLRP3 inflammasome. A fraction of glucose flux is metabolized by the hexosamine biosynthetic pathway to synthesize UDP-GlcNAc. OGT uses UDP-GlcNAc to O-GlcNAcylate TxNIP. This modification increases TxNIP-NLRP3 interaction. The formation of the TxNIP-NLRP3-ASC oligomer complex activates Caspase 1. Once activated, Caspase 1 cleaves the inactive form pro-IL1β to the biologically active form IL1β.

In conclusion, our study reveals that O-GlcNAcylation represents an important regulatory mechanism for TxNIP activity in β cells by increasing its interaction with NLRP3 and the subsequent stimulation of IL1β production (Figure 5). Additional studies will be required to identify TxNIP O-GlcNAcylation sites, and to establish the specific contribution of TxNIP O-GlcNAcylation to pancreatic glucotoxicity in diabetes. Overall, strategies to inhibit this novel regulatory node in pancreatic β cells could be of interest to limit inflammation in the context of hyperglycemia.

## Ethics Statement

Procedures were carried out according to the French guidelines for the care and use of experimental animals (validated by the Paris Descartes Ethical Committee).

## Author Contributions

FB and CP wrote the manuscript. GF, FB, PP, YF, and TI designed experiments, performed experiments, and analyzed the data. AI and JM prepared and provided islets from GK rats. CB, JK-C, and FP prepared and provided human islets. A-FB, SG, and TI provided critical comments on experimental design and on the manuscript.

### Conflict of Interest Statement

The authors declare that the research was conducted in the absence of any commercial or financial relationships that could be construed as a potential conflict of interest.

## Supplementary Material

Supplementary Figure 1 Transcriptional regulation of the *Txnip* promoter in response to glucose and OGA expression **(A)**. Schematic representation of the *Txnip* promoter (1081 base pairs; the two ChREBP binding sites (ChoRE) and the FoxO1 binding site (IRE) are indicated). **(B)** INS1 832/13 cells were infected by GFP or OGA adenovirus for 24 h. INS1 832/13 cells were then stimulated for 24 h under low glucose (2.5 mM) or high glucose (20 mM) concentrations. Luciferase activity of the *Txnip* promoter was measured. Figure is presented as means ± SEM from 3 to 4 independent cultures. Significance is based on two-way ANOVA followed by a Bonferroni *post-hoc* test. ^**^*p* < 0.01 when compared to GFP conditions, ^***^*p* < 0.005 when compared to 5 mM glucose condition (GFP), ^*$$*^*p* < 0.01 when compared to 20 mM glucose condition (GFP).

Supplemental Figure 2 INS1 832/13 cells were transfected with a BRET-based biosensor that monitors pro-Il1β cleavage. The histogram shows the decreased in BRET signal measured in INS1 832/13 cells after 24 h of incubation with PUGNAc (100 μM) or with ThiametG (10 μM). Significance is based on two-way ANOVA followed by a Dunnett's test for BRET experiments. ^**^*p* < 0.01 (*n* = 4).

## Abbreviations

ChoRE: carbohydrate response element
ChREBP: carbohydrate responsive element binding protein
FoxO1: forkhead boxO1 transcription factor
GK: Goto-Kakizaki
IGFBP1: insulin-like growth factor-binding protein 1
IL1β: interleukin 1β
NLRP3: NOD-like receptor family pyrin domain containing 3
OGA: O-GlcNAcase
O-GlcNAc: O-linked N-acetylglucosamine
OGT: O-GlcNAc transferase
qPCR: quantitative real time PCR
ROS: reactive oxygen species
TxNIP: thioredoxin-interacting protein
BRET: bioluminescence resonance energy transfer.
