# Supplementary figures and images for: O-GlcNacylation Links TxNIP to Inflammasome Activation in Pancreatic β Cells
Source: Front Endocrinol (Lausanne). 2019 May 21;10:291. doi: 10.3389/fendo.2019.00291 (PMC6536593; doi:10.3389/fendo.2019.00291)

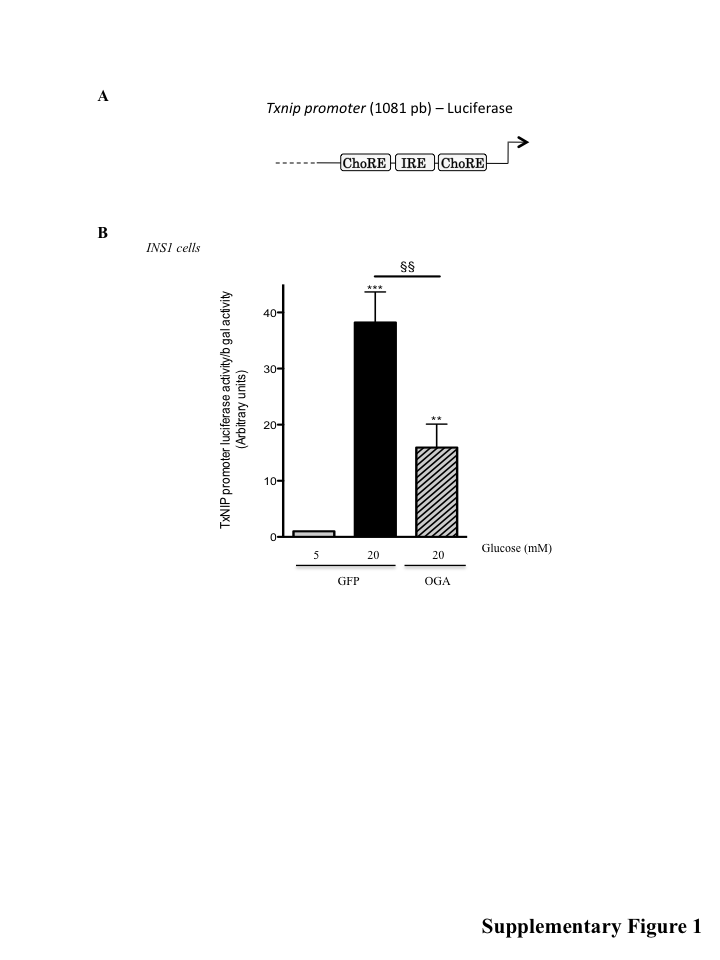

Supplement: Supplementary Figure 1 — Transcriptional regulation of the Txnip promoter in response to glucose and OGA expression (A). Schematic representation of the Txnip promoter (1081 base pairs; the two ChREBP binding sites (ChoRE) and the FoxO1 binding site (IRE) are indicated). (B) INS1 832/13 cells were infected by GFP or OGA adenovirus for 24 h. INS1 832/13 cells were then stimulated for 24 h under low glucose (2.5 mM) or high glucose (20 mM) concentrations. Luciferase activity of the Txnip promoter was measured. Figure is presented as means ± SEM from 3 to 4 independent cultures. Significance is based on two-way ANOVA followed by a Bonferroni post-hoc test. **p < 0.01 when compared to GFP conditions, ***p < 0.005 when compared to 5 mM glucose condition (GFP), $$p < 0.01 when compared to 20 mM glucose condition (GFP). [file Image_1.TIFF]

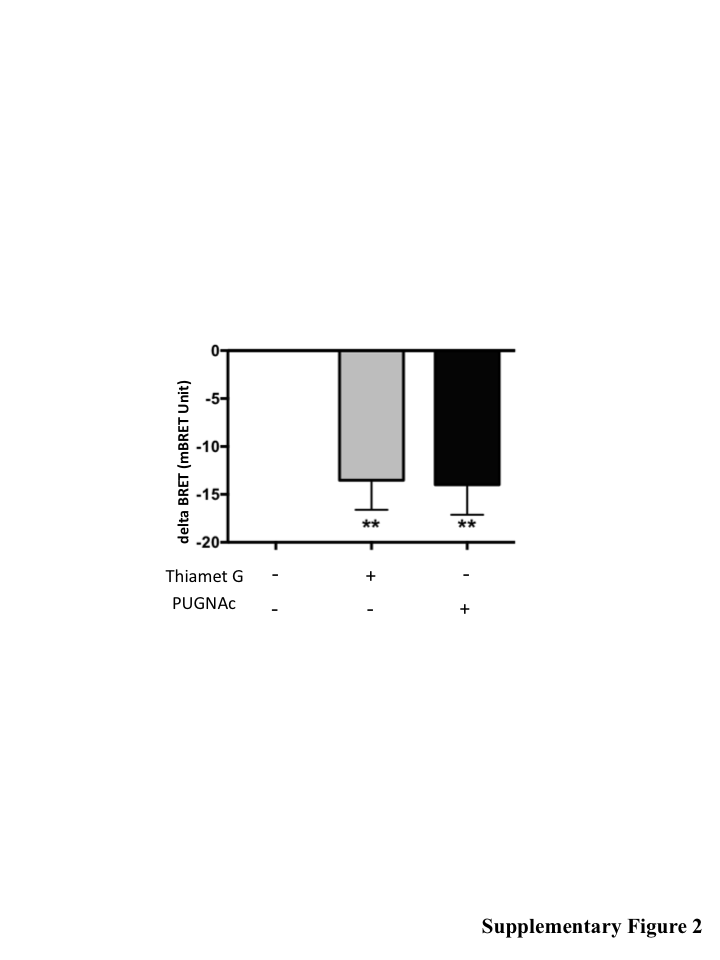

Supplement: Supplemental Figure 2 — INS1 832/13 cells were transfected with a BRET-based biosensor that monitors pro-Il1β cleavage. The histogram shows the decreased in BRET signal measured in INS1 832/13 cells after 24 h of incubation with PUGNAc (100 μM) or with ThiametG (10 μM). Significance is based on two-way ANOVA followed by a Dunnett's test for BRET experiments. **p < 0.01 (n = 4). [file Image_2.TIFF]
